# Brain is modulated by neuronal plasticity during postnatal development

**DOI:** 10.1186/s12576-021-00819-9

**Published:** 2021-11-17

**Authors:** Masoumeh Kourosh-Arami, Nasrin Hosseini, Alireza Komaki

**Affiliations:** 1 Department of Neuroscience, School of Advanced Technologies in Medicine, Iran University of Medical Sciences, Tehran, Iran; 2 Neuroscience Research Center, Iran University of Medical Sciences, Tehran, Iran; 3 Neurophysiology Research Center, Hamadan University of Medical Sciences, Hamadan, Iran

**Keywords:** Homosynaptic plasticity, Heterosynaptic plasticity, Excitatory synapses, Inhibitory synapses, Brain development

## Abstract

Neuroplasticity is referred to the ability of the nervous system to change its structure or functions as a result of former stimuli. It is a plausible mechanism underlying a dynamic brain through adaptation processes of neural structure and activity patterns. Nevertheless, it is still unclear how the plastic neural systems achieve and maintain their equilibrium. Additionally, the alterations of balanced brain dynamics under different plasticity rules have not been explored either. Therefore, the present article primarily aims to review recent research studies regarding homosynaptic and heterosynaptic neuroplasticity characterized by the manipulation of excitatory and inhibitory synaptic inputs. Moreover, it attempts to understand different mechanisms related to the main forms of synaptic plasticity at the excitatory and inhibitory synapses during the brain development processes. Hence, this study comprised surveying those articles published since 1988 and available through PubMed, Google Scholar and science direct databases on a keyword-based search paradigm. All in all, the study results presented extensive and corroborative pieces of evidence for the main types of plasticity, including the long-term potentiation (LTP) and long-term depression (LTD) of the excitatory and inhibitory postsynaptic potentials (EPSPs and IPSPs).

## Introduction

Plasticity is referred to the ability of an individual organism or cell in adjusting its phenotype in response to its environmental alterations. In contrast to prior views, recent studies have highlighted the extraordinary plasticity of cells [1]. Plasticity is a common synaptic feature. Accordingly, disclosing the molecular and cellular mechanisms that lead to this phenomenon is a dynamic biology domain with promising therapeutic potentials. Neuroplasticity, otherwise known as brain plasticity or neural plasticity, is the capacity of the neural synapses and brain pathways to be modified by altered thoughts and emotions, as well as environmental, behavioral, and neural stimuli. These repeated modifications occur as the brain learns and retains new data during its development [2]. Synaptic pruning usually happens when the brain deletes unnecessary or useless neural connections; this process simultaneously reinforces the necessary synapses [3]. Generally, the reformations of the synaptic network are experience-dependent processes in which the nervous system fine-tunes itself for competence. Moreover, its restructuring could provoke physiological and anatomical changes. For instance, the brain activity associated with a particular function could be relocated in the brain [4]. Nevertheless, important progress has been achieved in recognizing the molecular mechanisms of the elementary plasticity processes. However, the necessity and adequacy of synaptic plasticity in rearranging dynamic cortical developments cannot be easily demonstrated.

In this review conducted based on selected articles data extraction, and will be firstly discussed, homosynaptic and heterosynaptic plasticity and then the synaptic plasticity reinforcement and depression processes. Also, the key synaptic plasticity mechanisms, including the effects of development, synapse type, brain regions, and dendrite biophysics, as well as the postsynaptic changes occurring at excitatory glutamatergic synapses on locus coeruleus neurons would be explored. Finally, differential expression of long-term plasticity will be reported. Methods for developing this review are outlined in Box 1.

Box 1 Methods We identified articles from PubMed, Science Direct, and Google Scholar using the key search terms “synaptic plasticity” and “heterosynaptic”, “homosynaptic” from 1980 to the present. In total, 1100 related articles were found, of which 212 were on inclusion criteria (full original articles, review articles, books, and any scientific published data). The exclusion criteria were abstract or conference papers.

## Homosynaptic and heterosynaptic plasticity

Two plasticity types, homosynaptic and heterosynaptic ones, differ extensively in their necessity and respective presynaptic activity-dependency during the induction phase. Homosynaptic plasticity has shown whole-cell properties of neurons; however, heterosynaptic modulation remains restricted to individual synapses. Nevertheless, both processes interact at the level of single synapses [5, 6].

The Hebbian theory introduces three characteristics about synapses: homosynaptic plasticity, associativity, and input-specificity [7]. Accordingly, homosynaptic plasticity referred to as input-specific or associative plasticity, is induced at the directly activated synapses in a neuron during the brain’s developmental phase. To induce this type of plasticity at certain synapses, their presynaptic activation is required because they connect the postsynaptic neural firing to specific presynaptic neural activities [1].

In contrast to Heb theory, Kandel and Tauc [8] proposed a heterosynaptic rule for strengthening the synaptic connections. Experimental pieces of evidence have introduced the properties that were associated with heterosynaptic plasticity, including its induction at non-active synapses, weight-dependent direction and magnitude, and balanced potentiation and depression [9]. Therefore, it could occur at any cellular synapses following strong postsynaptic activities. Some differences between these two kinds of plasticity are shown in the following:

### Effect of plasticity on learning and memory

The heterosynaptic plasticity have been involved in enhanced learning and relearning capacity, as well as the increased spreading of inputs with intrinsic connections of the neural network [10]. This form of plasticity may play a key role in maintaining the ability to learn various tasks and developmental processes [11]. In dead, heterosynaptic plasticity is necessary for the formation, refinement, and/or modification of intrinsic connectivity, as well as the development of response selectivity [11].

Evidence has revealed that some behavioral learning processes, like classical conditioning and sensitization, occur after a certain stimulus input [12]. Although the non-associative heterosynaptic modulation holds purely heterosynaptic properties, the associative type is activity-dependent due to the combined features of homosynaptic and heterosynaptic mechanisms [8]. Homosynaptic and heterosynaptic types of plasticity may both contribute to memory and learning processes, mainly by modifying the potency of neural connections. Since these two forms of plasticity have different types of computational properties, they affect learning differently. They have different properties and supply different functions, but they can both be provoked by classical protocols of inducing plasticity [13]. The input-specific properties of homosynaptic plasticity lead to changes in the synaptic strength, occurring only at specific postsynaptic neurons that are already stimulated and activated [7, 14]. By contrast, in heterosynaptic plasticity, specific neural stimulations lead to non-specific input alterations in the synaptic weight [5]. At times, their alterations are complementary forms of plasticity; hence, they are both required for normal neural actions in synaptic plasticity [15].

### Duration of plasticity-induced alterations

Previously, Hebb [16] hypothesized homosynaptic rules for the long-term memory mechanisms; in this theory, those events that triggered synaptic reinforcement were proposed to have occurred at the same strengthened synapses [16]. According to the Hebbian hypothesis of homosynaptic plasticity, this process can always produce some distinct and short-term synaptic changes that cannot support long-term memory storage [5]. Therefore, the proposed mechanism might be used to explain learning and short-term memory; however, it may not recruit the required signaling pathways or transcriptional events for synaptic growth and long-term memory maintenance. Conversely, heterosynaptic facilitation could cause persistent changes when presented repeatedly by the transcriptional induction and new synaptic connections [5]. Also, the Hebbian homosynaptic and heterosynaptic modulatory mechanisms could recruit together in behavioral patterns [5]. Nevertheless, new synaptic plasticity categories could form due to their combination. Such joint mechanisms increase the duration of plastic changes in a non-additive way. Therefore, a greater level of synaptic specificity would be implicated that expands the nervous system’s ability to encode information [5]. Following the induction of homosynaptic changes, heterosynaptic plasticity was seen to be a common property of plastic synapses in the nervous systems. Heterosynaptic plasticity includes the neurons with operational stability that allow repetitive learning as well as the activation of dynamic features in sensory inputs [10]. In other words, the heterosynaptic changes may depend on postsynaptic firing and could associate with the homosynaptic plasticity induction; therefore, these changes demonstrate the intrinsic properties of synaptic plasticity.

Research studies indicate that Hebbian homosynaptic plasticity needs some modulatory transmitters to cause persistent changes. Also, homosynaptic action alone has not been sufficient to induce long-lasting plasticity. For instance, both homosynaptic and heterosynaptic processes are involved in classical conditioning. In conditional stimulus, the modulator neurons would release 5-HT with action on stimulated sensory neurons and undergo homosynaptic activity; then, the calcium influx into the sensory neurons increases the capability of 5-HT to activate adenylyl cyclase. Therefore, the temporal matching of heterosynaptic and homosynaptic activities causes an intense increase in cAMP concentrations and synaptic strength. Interestingly, these heterosynaptic and homosynaptic mechanisms have synergic effects. Therefore, the overall increase would be higher than the sum of both enhancements due to either heterosynaptic or homosynaptic processes alone. This event could be presented as a new plasticity class [5, 17, 18]: A combinatory mechanism that leads to a prolonged plasticity duration and ample synaptic specificity [5]. Additionally, the metaplastic effects of these combined mechanisms are associated with a new form of heterosynaptic synaptic depression, in which postsynaptic neural activity is simultaneous with weakened synaptic connections at the inactive synapses [19].

### Distance-dependency of plasticity

Distance-dependency of heterosynaptic plasticity (from the stimulated synapses during the induction) leads to specific changes in its amplitude; the same/opposite-sign plasticity would, respectively, occur at shorter/longer distances [9, 20]. Moreover, this amplitude alteration pattern may cause lateral inhibitions at synapses. Due to this class of inhibitions, plasticity occurs at a local synaptic population whereas it may stimulate other synaptic populations against the neighboring ones. Moreover, the total synaptic weight would be preserved to a cell by balancing the homosynaptic potentiation or depression [21]. Heterosynaptic plasticity could also be induced by distance-independent mechanisms, without presynaptic stimulations, and by the increase in intracellular Ca^2+^ levels (evoked by photolytic release of Ca^2+^ reserves) [22, 23]. Nonetheless, heterosynaptic potentiation or depression does not have identical induction rules [24]. Apart from the distance-dependency of the activation sites during the plasticity induction, the homosynaptic plasticity sign is a contributory factor as well. The same-sign heterosynaptic plasticity is induced at shorter distances while the opposite one appears farther away from the focal activation point [24].

### Plasticity latency

Overall, heterosynaptic and homosynaptic forms of plasticity (opposite terms) include different action durations. Homosynaptic plasticity has been reported to have needed 10 min for pairing, whereas heterosynaptic plasticity occurred 10–20 min after the pairing. The longer latency of heterosynaptic plasticity suggests that unpaired input changes serve as homeostatic modulators in synaptic exhaustion. However, homosynaptic plasticity is essential for the high performance of neural circuits [1]. While heterosynaptic plasticity is inhibited, homosynaptic plasticity could be preserved; therefore, heterosynaptic plasticity can exist in a non-stimulated pathway while a neighboring pathway is being stimulated [25].

Homosynaptic or heterosynaptic plasticity (Hebbian-type learning) characteristics and signal transduction are shown in Table 1.

**Table 1:** Homosynaptic inhibitory plasticity (Hebbian-type learning) and heterosynaptic inhibitory plasticity (non-associative plasticity [60, 70, 184]) characteristics and signal transduction

Heterosynaptic inhibitory plasticity (non-associative)	Homosynaptic inhibitory plasticity	References
Characteristics		
Occurs at active synapses)presynaptic activation(	Occurs at inactive synapses of homosynaptic plasticity	[52, 54–59, 62, 68, 69, 185–210]
Activity-dependent	Activity-independent	
Input specific	Not input specific	
Mediates associative modifications of synaptic weights	Affects a larger population of synapses	
Affects a larger population of synapses	Weight-dependent for amplitude of changes and direction of changes	
Related to short-term synaptic changes	Dependent on the distance from the site of induction of homosynaptic plasticity (Mexican hat like profile of amplitude)	
Persists for one or more hours		
Necessary for establishing and fine-tuning neuronal connections		
Induction protocols		
Episodes of strong postsynaptic activity at not active synapses	High or low-frequency stimulation by:	
Afferent tetanization	Afferent tetanization	
Pairing stimulations	Pairing stimulations	
Intracellular tetanization	Intracellular tetanization (purely postsynaptic stimulation)	
Plasticity type		
LTP, LTD	LTP, LTD	
Brain areas		
Involves cortical (hippocampus, visual cortex, ventral tegmental area) and subcortical area (deep cerebellar nuclei)	Involves cortical (neonatal and adult hippocampus, ventral tegmental) area	
Receptors		
VGCC	VGCC	
Both AMPAR and NMDAR	Both NMDAR-dependent and non-NMDAR- types	
mGluR I	Both mGlu1 and mGlu_2_	
GABA_A_Rs	Both GABA_A_R and GABA_B_ R	
D_2_R	D_1_R	
5-HT	α_1_ receptors	
*α* receptors	M_1_AChRs	
nAChRs		
CB_1_R		
Signaling molecule		
Ca^2+^	Ca^2+^	
IP3		
PLC		
DG		
NO		
Signaling pathway		
BDNF/TrkB	BDNF/TrkB	
cGMP- GC, PKG	cAMP-PKA	
cAMP⁄PKA	IP3	
βFGF	Sp-cAMPS	
∆FosB,	PKC	
CREB?		

### Homeostatic effects of plasticity

Several forms of heterosynaptic plasticity are reported, among which the main form has a homeostatic role [25]. The ultra-structural aspects, such as the synapse size and surface area of the postsynaptic density (PSD) could represent homeostatic regulations. The coordinated changes of the PSD surface area in the hippocampal dendritic spines after LTP induction can be mentioned as an example. The increased PSD surface area at some synapses and formation of new synapses have been accompanied by corresponding changes in the PSD surface area at other synapses. Whether it was a compensatory decrease or complete elimination, the total amount of PSD surface area stays approximately constant. Similar rules could be seen at individual dendritic branches as well [26–28].

## Region-specific plasticity in the brain

Different areas of the brain and nervous system could induce several forms of plasticity with a similar biological or experimental induction paradigm. Similar spike-timing-dependent plasticity (STDP) is a biological process that modulates the neural synaptic strength in the brain. This can lead to bidirectional corticostriatal (CS) and thalamostriatal (TS) STDP as anti-Hebbian CS-STDP and Hebbian TS-STDP [29] in physiological conditions without blocking the GABAergic transmission in the dorsolateral striatum [30–33].

In the somatosensory cortex, the deafferentation changes of capsaicin-induced C-fiber and the consequent peripheral inputs could cause cortical plasticity that would have been postsynaptic originally [34]. The electrophysiological analyses of nucleus tractus solitarii (NTS) neurons in the brainstem displayed hypertension-induced plasticity of GABAergic mechanisms [35]. In the raphe region of the brainstem, involved in cutaneous vasoconstriction due to hypothermia [36, 37], spatiotemporal developments and neural plasticity alterations occur in the serotonergic nuclei [38]. In another brainstem region, NTS neuroplasticity precedes the functional alterations in the autonomous adjustment of the arterial pressure [39].

Furthermore, the impact of thalamostriatal activity (through heterosynaptic plasticity) on shaping the corticostriatal plasticity maps in particular time scales could be significant. This heterosynaptic plasticity has a major role in shaping the corticostriatal plasticity map through the parafascicular thalamic nucleus (Pf) as well as the formation of flexible behaviors in procedural learning. Additionally, heterosynaptic plasticity at corticostriatal and thalamostriatal synapses has a significant impact on these plasticity maps. The slight precedence of cortical activation over the thalamic one or their simultaneous activation can either impose plasticity or disrupt corticostriatal plasticity. Also, thalamic inputs might strongly be modulated in corticostriatal plasticity maps through the heterosynaptic effects for specific timing patterns [29].

Certain signaling pathways, underlying the CS-STDP and TS-STDP, distinctively control the GABA levels. Moreover, the TS-STDP requires single molecular coincidence detectors (e.g., NMDA receptors or NMDARs), whereas CS-STDP needs both NMDARs and endocannabinoids (ECs) as distinct signaling pathways [33, 40, 41]. In this regard, there is evidence of inhibited GABAergic transmission in these excitatory synapses affecting the CS-STDP/TS-STDP polarity, and changing the bidirectional Hebbian TS-STDP to unidirectional anti-Hebbian STDP with LTD for the post–pre/pre–post pairings [29, 40]. At last, acetylcholine exerted a key role in the expression and polarity of both hippocampal and cortical NMDAR-mediated STDP; thus, the impacts of other neurotransmitters/modulators on similar mapping patterns need to be further explored [42, 43].

### Homosynaptic and heterosynaptic plasticity mechanisms in different brain areas

Different brain areas have different mechanisms for homosynaptic or heterosynaptic plasticity; for example, homosynaptic and heterosynaptic forms of plasticity in the mouse auditory cortex and human temporal lobe of epileptic patients displayed different mechanisms. Moreover, in the intercalated neurons of the amygdala, synaptic potentiation in a pathway can result in the depression of non-stimulated pathways [9].

Conversely, the cortical and hippocampal neurons can express a different form of plasticity, known as homosynaptic inhibitory plasticity (LTPi or LTDi), which is observed in some brain areas and circuit development [44]. The plasticity of GABAergic synapses from an individual inhibitory neuron onto a postsynaptic excitatory one is a homosynaptic monosynaptic form of inhibitory plasticity [45, 46]. The induction and expression of this form of plasticity exhibit significant differences in the hippocampus and sensory neocortex [44].

Sensory information is primarily conveyed to layers 3, 4, and 6 [47] of the neocortex via thalamocortical axons. The response latency to sensory stimuli is distinguished in layer 4 neurons [48, 49]. The sensory information principally flows through layer 4 to layers 2/3, and then to layers 5–6 [50], or through layer 4 to layers 2/3/5, and then to layer 6 [48]. The layer classifications in the somatosensory cortex of rats and monkeys, as well as the visual cortex of cats, correspond with the size of their receptive fields as follows: layer 4 (the smallest one), supragranular layers, layer 3, and infragranular layers [48]. Occasionally, the sizes of layer 3 and infragranular layers are equal to the ones in the supragranular layers [51]. Neurons gather information from other neurons at the previous level with larger receptive fields and deviate them to the next level. In this way, larger and more integrated receptive fields are formed.

Homosynaptic LTPi and LTDi both depend on postsynaptic Ca^2+^ currents. Nevertheless, the Ca^2+^ influx sources and their mechanisms have not been thoroughly explored yet [46]. Unlike layer 5 of the primary visual cortex or hippocampus, this form of inhibitory plasticity does not seem to depend on the changes in potassium chloride cotransporter 2 (KCC2) activity or the activation of either GABAB receptors or NMDA ones [46].

The induction and expression of high-frequency LTPi in the visual cortex are dependent on intracellular Ca^2+^ storage, which is triggered by the activation of GABA_B_ receptors. They are facilitated by the activation of serotoninergic (5-HT) or α-adrenergic receptors [52] and mediated through activating IP3 [53]. GABA release is mediated through a brain-derived neurotrophic factor and tropomyosin receptor kinase B (BDNF/TrkB) signaling cascade that is initiated by an intracellular Ca^2+^ release in the developing visual cortex [54] and hippocampus [55]; wherein, the high-frequency LTPi would be expressed presynaptically [44]. The maintenance of high-frequency LTPi in the visual cortex depends on persistent low-frequency stimulations (LFS). However, in the hippocampus, it is induced and maintained after the high-frequency stimulation (HFS) [56]. The ventral tegmental area (VTA) has a different mechanism. Its retrograde signaling pathways are mediated by nitric oxide (NO), guanylate cyclase (GC), and protein kinase G (PKG)-dependent pathways [57].

Also, the induction and expression mechanisms of heterosynaptic LTDi (long-term depression of IPSPs) illustrate significant differences in various circuits. For example, in layer 5 of the primary visual cortex, high-frequency LTDi depends on Ca2 + currents through NMDARs or L-type Ca2 + channels in postsynaptic excitatory neurons [58, 59]. The NMDAR-dependent LTDi produces a focal and restricted inhibitory depression, while the L-type Ca^2+^ channel-dependent LTDi depresses many inhibitory synapses that are related to the same postsynaptic neurons [58]. The ECs are also required for the high-frequency LTDi induction in layers 2/3 of the primary visual cortex and hippocampus [60].

The heterosynaptic LTPi of inhibitory postsynaptic potentials (IPSPs), has similar Ca^2+^-mediating signaling; nevertheless. However, it uses different sources of Ca^2+^ supply it has somewhat different underlying intracellular mechanisms for the induction and expression in the visual cortex [59], hippocampus [61], cerebellar nuclei [62], superior olivary complex [63], ventral tegmental area [64], brainstem [65], and other brain regions. For instance, the Ca^2+^ source is the voltage-gated calcium channels (VGCCs) in the neonatal hippocampus of rats [66], astrocytes in the young rat hippocampus [67], the postsynaptic intracellular reservoir for the visual cortical inhibitory synapses, and the postsynaptic NMDAR activation in the ventral tegmental area slices [68].

Moreover, low-frequency heterosynaptic LTDi has also been induced in several brain areas, including the VTA, amygdala, striatum, prefrontal cortex, and corticotectal cocultures [44]. Low-frequency LTDi is induced by the activation of glutamatergic axons, which can cause heterosynaptic depression in those GABAergic inputs that meet with the activated postsynaptic neurons and maintain their plasticity [69]. The EC release from the glutamatergic neurons to the postsynaptic ones affects the inhibitory synaptic strength [44]. The low-frequency LTDi requires the release and aggregation of Ca^2+^ in the presynaptic interneuron of the hippocampus. However, the presynaptic expression of low-frequency LTDi in the VTA occurs by GABA release in response to protein kinase A (PKA)-dependent modulations [60, 70].

Both homosynaptic and heterosynaptic inhibitory plasticity are involved in sensory processing, sound localization, neuropathic pain modulations, neural activity regulations after the brain injury, and pregnancy-induced neural excitability alterations [44]. Developing in-depth knowledge of different forms of plasticity is crucial to elucidate their role in brain functions in healthy subjects or the progression and treatment of diseases. Therefore, further investigations are necessary to identify their underlying mechanisms.

## Long-term potentiation and depression

Both LTP and LTD are involved in circuit and memory improvement in the developing sensory neocortex [71]. Overall, long-term plasticity depends on different variables, including the baseline amplitude of synaptic strength, presynaptic and postsynaptic spiking frequencies, postsynaptic membrane potentials, and the dendritic location of synaptic inputs [24, 72–74]. Some factors that affect the induction of LTP or LTD are as the following:

1. Different plasticity induction protocols have been used to define the direction and magnitude of homosynaptic plasticity and induce LTP and LTD, including afferent tetanization, pairing, and intracellular tetanization. The afferent tetanization is applied by simulating the presynaptic fibers using repeated electric pulses at a specified frequency or pattern with focal inputs that are decayed by distance. As such, low-frequency tetanization is given at 3 Hz and below, whereas high-frequency stimulation is received at 20 Hz and higher up to 50–200 Hz [24]. As such, in the afferent tetanization protocol, the change direction depends on the frequency. Therefore, the tetanic stimulations at higher frequencies (20 Hz and above) induce potentiation, but tetanization at lower frequencies (3 Hz and below) causes depression [24].
2. LTP or LTD induction mainly depends on the timing of presynaptic activity in the pairing protocol which relates to the postsynaptic firing or current network activities [24]. The LTP or LTD magnitude, however, is determined by the frequency and number of postsynaptic potentials in each pairing burst, as well as the number of pairings in the pairing protocol [72–74]. Any increase in these parameters results in higher alterations in plasticity [24]. Conclusively, high-frequency afferent tetanization induces a characteristic response amplitude profile containing alterations (LTP at stimulated inputs surrounded by heterosynaptic LTD in the hippocampus and amygdala) [9, 20].
3. Intracellular Ca^2+^ reserves play a major role in inducing heterosynaptic LTD and heterosynaptic LTP facilitation in the hippocampus [9]. Furthermore, inactive synapses have inverse sensitivity to local calcium signals [9]. Hence, higher levels of intracellular Ca^2+^ may lead to depression whereas the lower levels evoke potentiation at inactive synapses. This response profile corresponds with a hypothesis concerning the Ca^2+^-dependent LTP and LTD. Based on this evidence, the direction of synaptic alterations was related to the Ca^2+^ elevation amplitude and time course [75]. While fast and high-amplitude Ca^2+^ signals cause LTP induction, slow and low-amplitude signals could induce LTD. A brief and submicromolar increase in intracellular Ca^2+^ signals might lead to potentiation or depression changes [76].
4. Unlike heterosynaptic LTP, the LTD is partly mediated by a decrease in release probability. Although plasticity is regulated by presynaptic changes, it is induced by postsynaptic spiking alone. This form of plasticity would necessarily require transsynaptic interactions by the postsynaptic release of a retrograde messenger that activates presynaptic receptors following a strong postsynaptic depolarization [77].
5. Typically, LTP and LTD are produced by the postsynaptic activation of NMDA, α-amino-3-hydroxy-5-methyl-4-isoxazolepropionic acid (AMPA), and metabotropic glutamate (mGlu) receptors [78]. Among the molecules that underlie synaptic plasticity, AMPA-type ionotropic glutamatergic receptors (AMPARs) play a key role in both LTP [79, 80] and LTD [81]. In neurons, AMPARs are highly flexible and undergo essential and activity-dependent trafficking [82]. The changes in synaptic AMPAR number are crucial during the experience-dependent synaptic modulations. For instance, LTP in the pyramidal CA3 and CA1 hippocampal neurons is associated with an increase of synaptic AMPARs in an activity-dependent manner [83]. Conversely, the reduced number of synaptic AMPARs occurs in LTD. However, it is not clear whether such alterations in the AMPAR number at one synapse would also affect neighboring synapses in a compensatory manner. The modification of synaptic AMPARs during the LTP and LTD induction can provoke compensatory heterosynaptic alterations that could rescale the synaptic strength of unstimulated synapses and modulate consequent activity-depended synaptic plasticity inductions. In LTP and LTD, the increase or decrease of synaptic AMPARs highly depends on the lateral diffusion of receptors [84].
6. The interaction of retrograde messengers with specific receptors at the presynaptic membrane plays a role in inducing presynaptic LTP and LTD. In this way, the upregulation and downregulation of these receptors allow retrograde signaling to modify synaptic weight accordingly via certain proteins like protein kinase C (PKC) [85, 86]. Future expression of these postsynaptic receptors and the presynaptic receptors that correspond to them differ significantly concerning their development phases, synapse types, brain regions, and dendrite biophysics [78].

### LTP and LTD of excitatory synapses

The depolarization pairing with the CA3 presynaptic inputs in the CA1 neurons enhances the EPSP amplitude. This phenomenon is called LTP. Concurrent presynaptic and postsynaptic neural activities cause potentiation of synaptic conduction. Therefore, the excitatory synapses should contain coincidence-detector neurons to display synchronized presynaptic and postsynaptic neural activities. NMDA receptors are ligand-gated calcium channels that act as such detectors of presynaptic and postsynaptic depolarization [87]. The ensuing transient increase in the intracellular Ca^2+^ concentrations activates Ca^2+^/calmodulin-dependent protein kinase II (CAMKII) and PKC. Subsequently, the enzyme-catalyzed phosphorylation of cAMP-response element-binding protein (CREB) generates CREB-dependent gene expression [88]. Presynaptic terminals mediate LTP, and retrograde messengers, such as NO and ECs convey messages to the presynaptic cells so that neurotransmitter release could be altered [89].

Three procedures lead to LTP induction: (a) pairing, intracellular postsynaptic depolarization paired with the LFS of afferent fibers, (b) theta-burst stimulation (TBS) of the afferent pathways (10 brief bursts, 5 bursts/s; each burst four pulses at 100 Hz), and (c) tetanic stimulation (100 Hz, 1 s) of afferent pathways. The physiological relevance of these protocols may differ significantly.

LTP that is induced by tetanus [90, 91] and pairing stimulation of the white-matter (WM) [92] can be produced in pyramidal neurons in layers 2/3, 5, and 6 [93]. Some factors increase the likelihood of LTP production: blocking GABAergic inhibition, removal of Mg^2+^, or taking slices from immature animals [90, 91]. Decrease of inhibition and increase of excitation both enhances the probability of LTP induction. According to Kirkwood and Bear [90], LTP could be induced in layers‌ 2/3 by TBS of layer 4 with a success rate of over 80% [90]. Moreover, the visual cortex LTP mostly happens at synapses on layers 2/3, 4, and 5 [94, 95].

In a previous study by authors, corticogeniculate cells in layer 6 of the visual cortex received top-down synaptic inputs from cortical upper layers (Uls), and bottom-up synaptic inputs from the WM; also, the WM-induced and UL-induced plasticity could occur through NMDARs and mGluRs, respectively [93]. In LTD induction, repeated LFS reduces the synaptic efficacy [96]. However, in granular and agranular areas, homosynaptic LTD can be produced by layer 4 LFS (1 Hz for 15 min). LTD is induced by the LFS of the WM/layer 6 to layer 4 in immature animals in case of IPSP inhibition [97]. There is evidence that LTD in the visual cortex is mostly induced at synapses on layers 2/3, 4, and 5 [94, 95]. In our study regarding the CG cells of layer 6 (in the visual cortex), the cannabinoid type 1 receptors and calcineurin underlie the UL-induced and WM-induced heterosynaptic LTD, respectively. So, homosynaptic LTP and heterosynaptic LTD in corticogeniculate cells may modify the efficacy of synaptic transmission through different mechanisms [93].

### LTP and LTD of inhibitory synapses

The central and peripheral nervous systems include a variety of inhibitory interneurons [98]. In a mature nervous system, and especially in the adult cortical networks, excitation is modulated by a complex set of inhibitory circuits [99, 100]. Gamma-aminobutyric acid (GABA) is the main neurotransmitter that has major inhibitory functions. Inhibition is critical for many neural functions, including spike generation, dendritic integration, synaptic plasticity, sleeping, learning, and prevention of pathological activities like epilepsy [101–105].

Twenty-five percent of neocortical neurons are GABAergic [106], and 20% of all synapses are GABAergic [107]. Inhibitory plasticity may play a critical role in cortical remapping [108]. Besides, GABA receptors would be downregulated following the visual or somatosensory cortex deafferentation [109] whereas they would be upregulated by chronic stimuli [110]. To avoid hyperactivity or hypoactivity in neurons and nervous networks during prolonged periods, inhibitory synapses should be calibrated or balanced by the relative strength of excitatory synapses. In the sensory cortex, inhibitory responses and excitatory–inhibitory balance are developed in early postnatal developments [111]. Since the experience-dependent regulation of excitatory synapses mandates corresponding alterations to inhibition, the dynamic excitatory–inhibitory balance should be maintained as well [112–115]. Previous studies have implied the plasticity of excitatory synapses on inhibitory neurons, resulting in the discovery of highly heterogeneous rules for plasticity induction in diverse types of interneurons [116, 117]. It is indicated that some excitatory synapses on inhibitory neurons have shown associative Hebbian-type plasticity. As such, the interneural activities could present either input-specific or input-non-specific types of plasticity at different excitatory synapses [117]. For instance, excitatory synapses on fast-spiking (FS) interneurons of the stratum pyramidal cells lacked input-specificity in the hippocampal CA1 [116]. Contrastingly, the excitatory synapses on interneurons of the stratum radiatum and stratum oriens expressed strict input-specific plasticity. In the former instance, there is LTP expression but no LTD changes. In the latter instance, there is Hebbian-type plasticity in stratum radiatum or anti-Hebbian plasticity in the stratum oriens [118].

The chief inhibitory neurons in the neocortex may associate with one of the two common classes of interneurons, the FS and non-FS (nFS) neurons. They show different functions and properties [119]. Also, some excitatory synapses on the inhibitory neurons could induce heterosynaptic plasticity, with weight-dependent properties, both in FS and nFS subtypes and also in all interneurons that are pooled together, such as the pyramidal neurons [117, 120].

Recently, weight-dependent heterosynaptic plasticity has been proposed as a novel understanding of plasticity at the excitatory synapses on inhibitory neurons. It is a widespread phenomenon that may not only participate in preventing runaway dynamics at excitatory synapses, but also exhibit potentiation or depression dispositions [25, 117]. Despite the weight-dependency of heterosynaptic plasticity in all interneurons, it displays different net effects in the FS and nFS cells [117]. Heterosynaptic changes in the FS neurons would contribute to the overall excitatory/inhibitory balance. Also, they balance the cortical network operation patterns [121–123] while facilitating the local rearrangement of neural activities and their synchronization [117].

In the nFS neurons, heterosynaptic plasticity may contribute to the operation maintenance for the inhibitory systems by preventing the elimination of the low-probability synapses. Pruning prevention by Hebbian-type plasticity preserves the functional inhibitory neurons that were activated by low-probability synapses. That is because these synapses tend to be facilitatory for these neurons and may operate as slowly activated ones through repeated firing in the network. In addition to GABA, traces of NO involvement are found in retrograde signaling during the heterosynaptic plasticity induction in the pyramidal and inhibitory neurons [116, 124].

Recordings of the linked pyramid-to-interneuron pairs confirmed the possibility of plasticity induction by purely postsynaptic protocols without any presynaptic spikes; thus, the induced plasticity type was heterosynaptic [117]. At GABAergic synapses on the pyramidal neurons, tetanus stimulation of layer 4 in the visual cortex of adult rats [56] could induce plasticity in presence of both NMDA and AMPA receptor blockers. Unlike the associative EPSP potentiation, the IPSP plasticity was membrane-potential-independent.

In our previous study, in the layers 2/3 of the mouse visual cortex, the tetanic activation of presynaptic FS-GABA neurons induced LTP for the unitary IPSCs (uIPSCs); whereas a similar activation of presynaptic nFS-GABA neurons could not produce LTP. This evidence indicated that long-term plasticity at the inhibitory synapses on FS-GABA neurons has pathway specificity. Also, it proposed that P/Q-type calcium channels may involve in the LTP induction at inhibitory synapses on FS-GABA interneurons [125]. In another study, tetanic stimulation of the sensory cortex induced LTP in motor cortical neurons [126].

## Developmental plasticity

During postnatal developments in synaptic level, glutamatergic synapses become mature; also, various AMPAR and NMDAR activities occur. In the primary developmental stages, some brain synapses contain only NMDARs; therefore, the rapid addition of AMPARs into these synapses leads to their maturation. In our previous study, the postsynaptic alterations at excitatory glutamatergic synapses in the locus coeruleus of rats were analyzed to discover plasticity changes during the first postnatal weeks. The frequency and amplitude of NMDA sEPSCs and the frequency of AMPA sEPSCs in the locus coeruleus were increased during the second and third postnatal weeks compared to the first one [127]. Moreover, experience-dependent plasticity is a vital property of normal brain function that depends on regular LTP and is reduced in certain neurological and psychiatric disorders.

Experience-dependent plasticity can be mediated by the presynaptic NMDARs in the excitatory connections from the neurons of layer 4 and layers 2/3 in the visual cortex [128]. Also, the presynaptic NMDARs are involved in experience-dependent plasticity in excitatory connections from the neurons of layer 4 and layers 2/3 in the barrel cortex. However, in large excitatory spines of the CA1 hippocampal neurons, synapse-specific mediation of experience-dependent plasticity is facilitated by the activation of type-1 metabotropic glutamate receptors (mGluR1s) [128]. Synapse-type-specific variations of expressed proteins may be significant for synapse-type-specific plasticity (STSP); notably, proteins like CaMKII and mitogen-activated protein kinase (MAPK) are downstream signal pathways for neurotransmitter receptors [129]. However, postsynaptic signaling variations may control LTP. That is because the synapse-type-specific expression of the activity-related proteins like activity-regulated cytoskeleton-associated (ARC) proteins has affected homeostatic STSP [128].

According to previous pieces of evidence, enhancing NMDAR signaling could augment experience-dependent plasticity in the adult human brain [130].

Also, LTD in small immature dendritic spines only requires NMDAR activation, indicating the dynamic structure of dendritic spines in memory and cognition. However, in large mature dendritic spines, LTD needs activation of both NMDARs and mGluRs in addition to calcium release from internal reserves [131, 132]. Since large spines include more AMPARs [133], these findings correspond with metaplasticity, wherein previous activity could modify the following plasticity thresholds [134]. Sensory experience and neural activity adjust postsynaptic NMDAR subunit expression at many synapses of the brain [135].

Generally, age is a key factor in experience-dependent cortical plasticity. Significant alterations, as a result of stimulus-driven plasticity, occur primarily in critical stages of life [136]. These periods can later be revived according to a variety of elements, such as the damages of the peripheral sensory organs [136]. Such factors affect the plasticity changes in various life periods and do not function only in the critical periods of development. As such, these factors comprised myelin-associated proteins [137], inhibitory activities of parvalbumin-positive cells [115], and extracellular matrix components, including the perineuronal nets (PNNs) [136]. The number of parvalbumin (PV)- and somatostatin (SST)-positive interneurons deteriorates over time (as the subject ages). This indicates that different interneural subtypes would be affected differentially by aging. These results bear far-reaching consequences for developing rehabilitation plans targeted towards aging subjects [136].

## Biophysics of dendrites and their structural plasticity

Dendrites (or, dendrons) are distinct neural sites where action potentials (APs) occur [138]. Therefore, many studies have focused on how biophysics of dendritic affect synaptic plasticity. As such, pyramidal neurons comprised apical and basal dendrites, extensively branching to secondary, tertiary, and fourth-degree dendrites. These branching patterns physically restrict biochemical signal transmissions that serve as defining factors for the signaling pattern. Dendrites of pyramidal neurons contain dendritic spines, or tiny protrusions emanating from the dendrite surface [139].

In neocortical layer 5 pyramidal neurons, the distal excitatory synapses present less LTP than the proximal synapses do [129]. That is because the back-propagation of APs, which establishes potentiation in these neurons, cannot reach distal arbors [73]. Nevertheless, each synaptic connection is formed by various synaptic contacts at distal and proximal locations [140], indicating the possible region-specificity of plasticity. Therefore, while distal synapses may show LTD, proximal synapses could be potentiated.

In the hippocampal CA1 area, axonally coupled spine pairs on the same dendrite have a similar size; the ones on different dendrites have unidentical sizes [141, 142]. The strict correlation between the spine volume and synaptic strength [143] could be considered the evidence for input-specificity of plasticity even at individual levels of synaptic contacts [144].

Neural dendrites generally receive information from many synapses (approx. 10^3^–10^4^) and process the received information in milliseconds [145, 146]. Dendritic spines are tiny actin-rich, micronized protrusions projected out of the dendritic shafts. Any modification in the spine size is accompanied by synaptic strengthening at the level of an individual spine. Besides, the actin dynamics in the dendritic spine have important roles in structural plasticity [147–149].

Overall, the biochemical signal transduction needs a longer time to modify synaptic strength, dendritic excitability, and electrical information. Synaptic strength and electrical properties of dendrites, known as neural plasticity, are regulated by changes in signaling state. These changes are regulated by the ion channels and transmitter receptors [145, 146]. Moreover, the dendritic structures and properties have significant effects on framing the spatiotemporal patterns of signal transduction in biochemical information processing. For instance, the time course and spatial spreading of synaptic inputs are determined by the passive cable properties of dendrites along with the distribution and functional state of voltage-gated channels [150].

Long and thin dendrite are cable-like structures. Each of them has a conducting cytoplasmic core and plasma that have membrane surface area with resistance and capacitance [151–154], displaying cable properties. These properties include current flow along the length of cable and across the membrane, as well as the drop of voltage across the membrane [73]. Particularly, the decrease of voltage is seen in the subthreshold regime of the long and thin dendrites that are related to a large axial resistance [155]. The asymmetric functional structure of dendritic trees affects the transient and steady-state voltage attenuation of subthreshold signals. Also, it results in an asymmetric activation of back- and forward propagating spikes [73]. Accordingly, the EPSP amplitude peak is attenuated by propagation at sites of origin to the soma (over 100-fold for most distal synapses in the neocortical pyramidal neurons of layer 5) [156]. This dendritic voltage decrement causes synapses at different dendritic locations to be influential (although not equally) on axonal spike outputs [157]. However, the synaptic charge attenuation in long and thin dendrites of pyramidal cells significantly reduces the amplitude of somatic EPSP (originating from distally located synapses on dendrites), compared to the proximally generated EPSPs with the same synaptic conductance. In the short spiny branchlets of Purkinje cells that are directly connected to the main thick dendrites, equal synaptic conductance was simulated on distal and proximal spiny branchlets with a similar somatic EPSP amplitude [158, 159]. The cable equations imply that electrotonic conduction of a somatic depolarization cannot fully preserve the somatic steady-state or transient depolarization as synaptic or action potential of all dendrites [160–162]. This problem is resolved by employing high-threshold VDCCs and their associated Na^+^ currents, instead of NMDARs or Ca^+2^ current [163, 164].

The dendrites could act as resistive and capacitive types of load on the axonal spike-initiation sites, causing difficult AP initiation. Therefore, dendritic morphology has a powerful impact on the neural input–output (I/O) function [73]. As such, the considerable increase of membrane area leads to large capacitive load with severe amplitude attenuation of fast-transient voltage; thus, it leads to the quick drop of AP, below the normal threshold of active propagation [165, 166]. Dendritic morphology determines which associations could occur between different synaptic inputs or input–output during the synaptic integration and plasticity [73]. Dendritic morphology also alters the coupling between somatic and dendritic spike-initiation sites in neurons [73, 167].

In addition to dendritic morphology, other dendritic properties, such as the kinetics, density, and spatial distribution of various voltage-gated conduction play major roles in spreading synaptic potentials, back-propagation of APs, initiation conditions, and forward propagation of dendritic spikes [168]. Also, regulation of channel properties and density, as well as the spatial gradients in these variables are functionally important neural characteristics. Besides, different kinds of voltage-gated conduction exist at different dendritic tree locations that may selectively modify the excitability of different neural types [168].

Some studies have proved the role of dendrites in input–output transformation and long-term synaptic plasticity. Therefore, local dendritic responses are presented as important factors that have a decisive impact on the nature and outcomes of synaptic plasticity [169]. Moreover, the biochemical synaptic transmissions reach postsynaptic density (PSD) in the dendritic spine and integrate with synaptic transmission [139]. Certain structural properties of the dendritic spines, such as the PSD surface area or spine head volume, have, respectively, influenced and correlated with the changes of synaptic efficacy [170]. Since the total surface area of PSD is approximately constant, any increase in the synaptic PSD surface area (i.e., new synapses) is balanced by a corresponding decrease in the number of other synapses, or by their complete elimination [27]. For instance, spine head volume is directly related to the PSD size; or long-term enlargement of spine size is associated with the LTP of synaptic transmission, so that spine head volume, PSD, and postsynaptic sensitivity to glutamate could increase [171, 172]. Any rapid increase in the sensitivity of the spine head volume or postsynaptic sensitivity to glutamate happens within few minutes despite the slow increase (in approximately 1 h) of PSD volume [172–174]. Accordingly, an opposite mechanism is also proposed for weak and prolonged stimulation, such as LTD and spine shrinkage [175–177].

Intracellular processes, as well as the extracellular signaling in dendritic spines, play significant roles in synaptic plasticity. These spines release BDNF through Ca^2+^-CaMKII-dependency. The released BDNF is bound to its TrkB receptors on the same spine and activates the receptors’ signaling to GTPase proteins Rac1 and Cdc42 to regulate the actin. Moreover, the extracellular signal-regulated kinase (ERK) and PKA pathways are involved in protein synthesis regulation in the activated dendritic spines [178–181]. Other than signaling protein activation (through rapid spine shape modification), also, activity-dependent protein synthesis in dendrites is contributory to the synaptic plasticity maintenance for more than several hours. Also, some GTPase proteins, such as Ras, RhoA, and Rac1 are important in facilitating the spine plasticity of adjacent spines. In other words, they lead to cooperative synaptic plasticity in adjacent spines [182, 183].

Since both passive and active dendritic features influence the local integration and forward propagation of evoked potentials, the effects of certain inputs on the spike output are determined by them. These passive and active properties also play a major role in synaptic plasticity activation as they establish both electrical and chemical signals received at each synapse and the interactions between the synapses. At last, the features of the dendritic trees could be modulated. Therefore, the electrical properties of dendrites could provide a vast range of mechanisms for plasticity modulation [73].

## Conclusion

The homosynaptic and heterosynaptic plasticity are two key forms of plasticity that contribute to memory and learning processes with some differences in processes, mainly by modifying the potency of neural connections. They have different properties and supply different functions. Homosynaptic plasticity always produce some distinct and short-term synaptic changes that cannot support long-term memory storage, while heterosynaptic facilitation could cause persistent changes. The longer latency of heterosynaptic plasticity suggests that unpaired input changes serve as homeostatic modulators in synaptic exhaustion. However, homosynaptic plasticity is essential for the high performance of neural circuits. While heterosynaptic plasticity is inhibited, homosynaptic plasticity could be preserved; therefore, heterosynaptic plasticity can exist in a non-stimulated pathway while a neighboring pathway is being stimulated. In addition, different areas of the brain and nervous system are involved to form homo- and heterosynaptic plasticity. Heterosynaptic plasticity contributes to shaping the corticostriatal plasticity maps in particular time scales. This heterosynaptic plasticity has a major role in shaping the corticostriatal plasticity, and has a significant impact on the brain plasticity maps. Different brain areas have different mechanisms for homosynaptic or heterosynaptic plasticity that need to be more investigated and elucidated.

## Data Availability

Not applicable.
